# Editorial and Miscellaneous

**Published:** 1855-11

**Authors:** 


### EDITORIAL AND MISCELLANEOUS.

### OUR ENTERPRISE.

Upon the whole, we have no reason to be dissatisfied, or cause for complaint connected, with the reception of our Journal, either from the profession generally or from the press.

So far as any special notice has been taken of us, within our knowledge, with a single exception, we have received at least courtesy and justice. This is all we ask. In some instances we have had a manifestation of generosity and kindness, and for this we tender our acknowledgments.

Our Journal has been set afloat because we have been *satisfied* that there was a sufficient field for our operations, and one, as we believed, demanding all the efforts that could be brought to bear for the advancement of true medicine and arrest of the rapid inroads which quackery, in its multiplied forms, is known to have been making in this region of country.

In addition to this, we have believed it to be our interest, being confident that it would command a sufficient patronage to sustain it, determined, however, under any circumstances, to make it a permanent publication.

We have yet had no reason to regret its commencement. Our subscription list has been steadily increasing, and we have certainly no reason to believe that our subscribers are dissatisfied with the result of the effort to establish a respectable Medical Journal. That we have committed errors, and have reason to be dissatisfied with ourselves, is readily admitted; and that there will be a continuance in the failure to perform our *whole duty*, we do not doubt, and that the point of considering ourselves infallible will not be attained, we earnestly hope; and in this connection we have to say that, to be reminded of faults or omissions in the discharge of duty, in a proper manner, we do not object, but rather "covet as one of the best gifts" which we can receive.

To be subjected to just criticism is all right, and to be expected; but to be made the subject of unprovoked and unwarrantable attack, we cannot consent, without at least an effort to defend.

Our object is to be quiet and unobtrusive collaborators for the advancement of medical science and the alleviation of human woe, and if forced into controversy at all, to contend with those who are seeking to pull down our fair fabric, and who would glory in its crumbling dust, rather than with those whom we recognize as brethren, engaged in a common cause.

While we are not at all warlike ourselves, and prefer the peaceful presentation of science, as the most efficient means for its promotion, if there are belligerent spirits in our ranks, who are not satisfied without a declaration of war, surely there are acknowledged enemies enough against whom to direct their batteries, without turning them against friends; and if there are any of our seniors who may suppose their interests to conflict in any way with ours, we would say to them let there be no quarrel between us. If they consider themselves entitled to the front rank or the right wing, we will quietly and willingly, as juniors in service, take our places in the rear or on the left, or at whatever point our services are demanded or our merits should assign us. That there is a sufficient field and labor enough for all to perform, there is no doubt; let us, therefore, present a united band, laboring for the accomplishment of the great objects which we profess to have in view, and instead of wasting energy and bringing discredit upon ourselves in domestic strife, direct our efforts to the upbuilding of science. We do not base any of our hopes of success upon the ability to injure others, and it is no part of our design to attempt to interfere in any way with the interests of any, but rather strengthen all, to the extent that our humble labors may aid in the advancement of true medicine, and inducing a proper appreciation of its merits upon the part of the world.

---

WE have received "Letters to a Young Physician," by Dr. JAS. JACKSON, of Boston, from the publishers, Messrs. Phillips, Simpson & Co. It will be noticed more particularly in the next number of our Journal, having arrived too late for an examination before our present issue.

### "HONOR TO WHOM HONOR IS DUE."

Having set out in our enterprise upon the principle of "rendering unto Cæsar the things that are Cæsar's," we were no little surprised to find it stated in the last number of the Nashville Journal of Medicine and Surgery, under the head of "Medical Items prepared by the Assistant Editor for the October number," that we had so soon been guilty of a departure from what is certainly intended to be our rule of action, and we now hasten to make amends for our failure in this particular.

Be it known to the medical world that PAUL F. EVE, M. D., Professor of Surgery in the Nashville University, and Assistant Editor of the Nashville Journal of Medicine and Surgery, *claims* the following "*credit*:"

"We ask pardon of Dr. E. Harris for the alteration of the title, which should have been as he published it,* 'Asphyxia from Burning Gas,' in the New York Medical Times.

"The apology is this: we found the case so striking a one that we added the words, 'A very creditable Case—Life saved by the Doctor,' which our compositor unfortunately substituted for the original heading.

"This explanation is now the more necessary as we find the article has been copied from our Journal, *without credit*, with *our title* to it, in staring *capitals*, into the Atlanta Medical and Surgical Journal, and this change, ascribed to the author of the communication, places him in the unpleasant position of lauding his own doings."

*Our* apology to the aggrieved assistant editor is this: We did not *know* that he was the author of the *alteration of title*, for which he is so strenuously contending, until informed of the fact in the very courteous article above inserted; and upon an investigation of the matter, cannot see how we are chargeable with very culpable ignorance, as we had no means of ascertaining that this addition or alteration had been made by our *claimant*.

How could we tell whether the title originated with the New York Medical Times or with the Nashville Journal?

---

* "We believe, but have misplaced the Journal."

As to the author of the article being placed in the unpleasant position of lauding his own doings, if it were so, who is chargeable with it but the assistant editor, by *his own confession?* That it *did not*, however, originate with the author of the article is too manifest to admit of a moment's thought. This would have been evident to the mind of the most simple reader.

We credited the article to the New York Medical Times, in which Journal it originally appeared, and in this connection we would desire to know, if an article going the rounds of the host of medical journals in the country must be credited to the whole list through which it goes? If so, in many instances you would have your catalogue of journals exceeding in length the article to which they are attached.

We could have had no possible objection to attaching "Nashville Journal" to the article, if we had supposed that even courtesy required it; but if we had, how *this* would have relieved the editor from the gross carelessness to which he confesses, we cannot for our lives conceive; and that such a matter should have been made the occasion of an attack upon us, we consider to be simply and in plain terms, absurd, ungenerous and unjust.

We have commenced this Journal with very moderate expectations and pretensions, and really feel ourselves entitled at least to common courtesy at the hands of our editorial brethren, who are already in the full tide of prosperity; and at this point we feel disposed to acknowledge that in the regular editorial department of the very number containing this onslaught upon us, we have had all of justice and even of kindness manifested towards us which we could have desired, and are therefore *compelled* to the conclusion that the injustice of which we complain was an individual matter.

We all are, or should be, working for a common cause—the advancement of medical science and the promotion of good feeling among the profession, rather than the fostering of heartburnings and jealousy, which have been so long a disgrace to our calling, and it is only by brow-beating and unprovoked assaults that we shall be led off from the pacific course which we have laid out for ourselves.

THE following excellent sentiments in reference to the relative duties and obligations connected with our profession, are extracted from *Tanner's Clinical Medicine*, a small work which we noticed in the last number of our Journal, the value of which has increased, in our estimation, the more we have examined it:

*The General Conduct of the Medical Practitioner.*—Although much might be advantageously written upon this subject, yet a very few words must suffice. The mere fact that the practice of medicine arose from an instinctive impulse to relieve the pains and sufferings of others is sufficient to show that the medical man, of all men, should be free from that vice which is the besetting sin of mankind—selfishness. He must indeed be thoroughly content to live, not for himself, but for others; not to look to his own interests, not to be guided in his actions by motives of policy, but to let the rule of his life be to do as much good to others as possible. He should think as little of pecuniary rewards as is compatible with his own interests and that of his brother practitioners, remembering the maxim adopted by LaBruyere from Confucius—that he who esteems gold more than virtue, will be likely to lose both gold and virtue. The physician, to be successful, must not only possess a sound practical knowledge of his profession, but he must also be careful that his moral character be free from blemish; that his general conduct be not only above vulgarity, but such as to excite the respect of his friends and neighbors; that he be conscientious, attentive, careful of the secrets of those who consult him, unmindful of the worldly condition of his patients, sympathizing, calm, and circumspect in his behavior generally. As it is his object to prolong life, so he must leave no means unpursued in order to attain such object, remembering that the mere prescribing of medicines is often the least part of his duty. It would indeed be well if medical men generally thought more of the *moral* remedies at their disposal; and if more attention were bestowed upon soothing the fleeting moments of ihe afflicted, by inspiring them with hope, confidence, and ease of mind. A man who practises his profession conscientiously will never be unmindful of the duties which he owes to his colleagues—to those treading the same path as himself. He will carefully avoid all such short-sided proceedings as may tend to elevate himself by depressing others; he will strictly eschew those disgraceful methods of obtaining notoriety, newspaper puffing or prescribing; and he will hesitate at giving, as a rule, gratuitous advice, where such is not needed by the circumstances of the patient, and where such a course of proceeding must injure those who are content to receive a small remuneration for their toilsome labors, and whose daily bread probably depends upon their obtaining such a return for their exertions.

The encouragement bestowed upon medical men is for the most part very deficient, their worth and usefulness being unacknowledged, their fatigues and anxieties unheeded, and their unselfishness and disregard of wealth abused. While striving to diminish the sufferings of their afflicted fellow-creatures, can it happen otherwise than their feelings should be hurt by observing the attention paid to men practising the most palpable absurdities and deceptions, by witnessing the success of homœpaths, table-turners, mesmerists, and such like? Has it not, however, always been so? Does not Bacon himself tell us that 'the weakness and credulity of men is such, as they will often prefer a mountebank or witch before a learned physician,'* and is the present age less credulous than that of the great philosopher? I fear not! But it is the prerogative of superior minds to rise with the occasion. Let us, therefore, individually and collectively, as students and practitioners, strive to improve our art: let us each endeavor to attain that mental sagacity which will enable us to perceive the important features of cases coming under our care and the salient points of diagnosis; that wisdom which can foresee the course and progress of disease; that judgment which will enable us to select the proper remedies; and that calm determination which will render us capable of insisting that the necessary measures are thoroughly carried out.

---

### RHEUMATIC PNEUMONIA.

We have been particularly impressed by the article which we publish in this connection, from the fact, that some months since an esteemed clergyman and friend consulted us in reference to an affection of the throat, which had seriously impaired his voice, and had consequently excited great apprehension on his part as to the result. After its continuance for some weeks without any decided relief, and when he had been fearfully contemplating a cessation of labor as essential to his restoration, he became the subject of a sudden attack of rheumatism in the knee, attended with the almost immediate subsidence of the disease in the throat. He soon recovered from the secondary affection under appropriate treatment and has had no return of the disease in its primary seat, notwithstanding the continued and active use of his vocal organ in his regular calling. That the original affection was rheumatic, we are perfectly satisfied, and that the whole track of the respiratory apparatus may be the subject of similar disease, we have no doubt, and look upon the fact as connected with very important pathological and therapeutic enquiries.—EDS. ATLANTA MED. & SURG. JOURNAL.

*Rheumatic Pneumonia.*—About a month since, a young man was admitted with all the signs of pneumonia, and, kermes having been administered, the next day all traces of the pneumonia had disappeared. To what were we to attribute so sudden a retrocession? Was it the result of treatment, or must we seek for the cause in some peculiarities attaching to the nature of the disease itself? The latter interpretation received some light from what was observed at the next visit, when the left great toe was found red, swollen, and painful, the tendinous sheaths

---

* The Advancement of Learning.

along the dorsum of the foot exhibited a like condition. Next day, the right foot was similarly affected, though in a less degree. Two days ago, a woman was admitted with the following symptoms: strong febrile action, redness and swelling of the left leg and foot, and severe pain in the entire upper extremity and trunk of the same side, the pain exciting cries on moving the parts. The patient especially suffered at the left side of the chest, but no abnormal sounds were audible.—During the night, cough came on, and in the morning a manifest *souffle* was audible in the supra-spinal fossa, while around and in the infra-spinal fossa was heard a fine sub-crepitant *rale.* During the cough, dry crepitating *rale* and bronchophony were heard, and two or three pneumonic sputa were expelled. This morning all signs of pneumonia have vanished. Here again I hesitate to attribute such prompt resolution to the treatment, especially as the apex was the part involved—a form of pneumonia regarded by all physicians as especially serious. I prefer explaining so rapid a termination by the nature of the pneumonia itself, which I regard as *rheumatic.*

Too partial to localization, practitioners are only accustomed to recognize rheumatism as affecting certain tissues, viz: the muscles, the aponeuroses, and the joints, and when it manifests itself elsewhere they call it by some other name.—This is as if we only acknowledge syphilis as we observe it on the penis, and made so many distinct affections of its manifestations on the throat, skin, etc. But syphilis is recognized to be the disease in all these accidents, and why should it not be so with rheumatism? That it attacks all serous membranes is an indisputable fact since Bouillaud's beautiful researches, which have so much advanced the pathology of the heart, When in the course of acute articular rheumatism any of the serous membranes become affected, it is termed a pericarditis, pleuritis, meningitis, etc., according to the membrane attacked. This is right enough as far as it goes; but for the proper denomination of the disease, which is a kind of definition in a single word, we ought to add the epithet "rheumatic." When a man accustomed to suffer from rheumatism acquires, as a consequence of cold, a pain of the shoulder, hip, etc., he at once says he has an attack of rheumatism. But instead of this pain let there be a sore throat, and both patient and doctor cease to be logical, and call it angina instead of rheumatism; just as if there were not a true rheumatic pain of the fibrous parts of the pharynx and palate, pain followed by fluxion, tumefaction, and redness of pharyngeal mucous membrane. Do we not find rheumatism of the fibro-serous tissues of a joint accompanied by tumefaction of the subcutaneous cellular tissue, and bright redness of the skin; and why should we not admit the same influence in the delicate and vascular mucous membrane? For my part, I should not hesitate to recognize a rheumatism in such a case, or, if you like it better, a rheumatic angina.

This distinction may serve for the explanation of the very great differences observed in the progress and termination of anginas, regarded by some physicians as being of the same nature. Thus, simple inflammation of the tonsils goes through all its stages, in spite of whatever treatment may be opposed to it, and a patient accustomed to such attacks will warn his attendants of the inutility of endeavoring to prevent the formation of abscess. A rheumatic angina, on the contrary, will often disappear in the course of a night, whatever the treatment adopted, leaving the physician astonished at the therapeutical success, the result, however, being really due to the essentially mobile character of the affection. Descending lower down in the digestive canal, we can explain those sudden diarrhœas which manifest themselves under the influence of a chill. The fibrous portions of the canal become painful, and the contractions more considerable and more frequent, a fluxionary movement being at the same time established towards the mucous membrane, the secretions of which are increased. Such diarrhœas are of short duration, unless, indeed, the rheumatism takes on, as it may anywhere, a chronic character.

After these considerations, does it seem strange to admit a rheumatic pneumonia? Suppose the pulmonary tissue, or, what is the same thing, the fibrous tissue of the extremity of the bronchi, becomes seized with the rheumatism, what are the immediate results? Tumefaction and congestion of the mucous membrane and an infiltration of the cellular tissue; that is to say, the anatomo-pathological conditions of œdema or of pneumonia in its earliest stage; with this peculiarity, that such lesions, participating in the fugacious nature of rheumatism, do not possess the fixity and persistence of the lesions of ordinary puenmonia. It is in cases like these that therapeutical results seem so marvelous, and so they would in our own two cases had we not a better reason to give for the rapidity of the cure.—They were, in fact, the examples of rheumatic pneumonia, the one occurring in a young man who was at the same time suffering from rheumatism of both feet, and the other in a girl who had formerly had rheumatism, and together with the pain in the chest complained of rheumatic pains along the whole of the same side of the body. In similar cases, I shall not hesitate to admit the existence of rheumatic pneumonia, too happy only thus to complete my diagnosis, and to become enlightened as to the amount of importance that should properly attach to my therapeutics.—*London Lancet and New Orleans Medical and Hospital Gazette.*

---

We find the following interesting case in the *Boston Medical & Surgical Journal*, under the head of "Extracts from the Records of the Boston Society for Medical Information. By Wm. W. Morland, M. D., Secretary:"

*Paralysis supervening after Anæsthesia.*—Dr. Hooker, of East Cambridge, Associate member of the Society, reported the following case.

In the month of November, 1853, I administered sulphuric ether to a gentleman from the country, for the purpose of removing a fatty tumor from the back part of the left shoulder and neck. The tumor extended from a little above the spinous process of the scapula along the trapezius muscle upwards, to its insertion in the occiput. The tumor was wholly between the fascia covering the muscle and the skin, leaving but little adipose matter between the tumor and the skin. The tumor was confined wholly to the left side, not extending over the median line, and was from five to six inches in length, and from two to three inches in width. The operation and dressing did not occupy more than half an hour, and during that time about eight ounces of concentrated sulphuric ether were used. For only a short space of time was the patient completely narcotized. The operation was performed in the sitting posture, and but little blood was lost. Before the dressing was completed, a change came over him, resembling syncope, and he was removed to his bed. Free emesis then took place, and he soon rallied, but not to perfect consciousness. After a little delay, he was left for the night, apparently as comfortable as is usual after etherization.

The next morning he gave the following account of himself during the evening and night. As his consciousness returned, he found the same prickling and benumbed sensation in his limbs, that he felt creeping over him when he first began to inhale ether. This was alike on both sides of his body. It soon passed off from the left side, but did not from the right. During the night, he found that he could not move his right arm and right leg, that they were destitute of sensation, and that he had not been able to move himself from the position in bed in which we had placed him after the operation. In this condition I found him in the morning. Complete paralysis of the right side, excepting the nerves of the face, tongue and throat. He had retention of urine. The paralysis of the extremities continued for nearly a year, before he was able to use his limbs with any degree of freedom.

He was a man of 40 years, healthy, of abstemious habits—a rigid cold water drinker; had never had any paralysis before, nor is there any predisposition to it in the family. He was of a taciturn character, but for two or three weeks after the operation he was jocose, exhibiting a degree of levity entirely at variance with his usual turn of mind. He had taken a good deal of exercise in the early part of the day of the operation, and had eaten pretty freely of a boiled dinner, consisting of potatoes and squash, with a small amount of corned beef, as was shown by what he ejected from his stomach. It will be remembered that he did not vomit till after he was placed in bed, and after the syncope.

The interesting question now occurs, what was the cause of the paralysis? Was it in the inhaling of ether, or was it from the loaded state of the stomach and the shock the system may have sustained from the operation? To the friends, I suggested that it might have arisen from the two latter cases, as ether had, to my knowledge, never been known to produce permanent paralysis. I must confess, however, that the suggestion was far from being satisfactory to myself, and I have thought the case worthy of being reported to the profession.

[Sulphuric ether has been so uniformly safe in its operation on the system by inhalation, that it seems very unlikely the paralysis in the above case was due to its action. Certainly, were such the tendency, even in an infrequent degree, we should have heard of cases, by this time, among the thousands in which it has been employed to produce anæsthesia. So far as published reports testify, there are, we believe, none such on record, nor can we learn of any on inquiry. Any other cause should be assigned, in our opinion, in preference to the ether.—SECRETARY.]

### EXCHANGES.

Besides those already acknowledged, we have received the following publications, to a number of which we are indebted for flattering notices of our Journal: The Medical Chronicle or Montreal Monthly Journal of Medicine and Surgery; Nashville Journal of Medicine & Surgery; New Orleans Medical News & Hospital Gazette; Southern Medical & Surgical Journal, Augusta; Proceedings and Reports of the Medical & Chirurgical Faculty, of Maryland; American Journal of Insanity, Utica, New York; New York Scalpel; Medical Examiner, Philadelphia.

### OMISSION.

The compositor inadvertently failed to credit Symes' Clinical Lecture, which appeared in our last number, to the *London Lancet.* This is a point upon which we have desired to be very particular, and whenever there occurs any failure to do justice to all, it will be a source of regret and an object to make prompt reparation.

---

*Premature Labor.*—The Boston Medical and Surgical Journal contains an article on the subject of inducing premature labor, which is to form a part of the forthcoming work of Professor Simpson. Various plans have been used for this object, all of which are successful, but preferences are given to some over others, on account of their giving less pain and inconvenience to the mother, and affording greater security for the life of the child. Several of these plans, once in vogue, have now been abandoned; namely, abdominal frictions, electricity, counter-irritation, ergot and other ecbolic medicines, rupture of the membranes, &c. Preference is given to dilatation of the os uteri, which may be done by means of the finger or sponge tents, the latter being the least painful; to injections of warm water into the vagina, or into the uterine cavity, frequently repeated; and to the separation of the membranes from the cavity of the cervix or body of the uterus by the finger, or by the uterine sound. After trying various plans, Prof. Simpson now prefers that by the sound or uterine bougie, which he passes upward, between the membranes and the anterior wall of the uterus for five or six inches. This gives little or no pain to the mother, and affords the best chance of life to the fetus, by retaining the liquor amnii until the labor is far advanced. It is thought to be desirable to avoid the detachment of any part of the placenta, the actual position of which may be previously ascertained by means of auscultation. In most cases, however, the sound has to be introduced more than once, and sometimes it has been necessary to follow its use, by injections of warm water into the uterine cavity. All the children have been born alive in the ten or twelve cases in which he has induced premature labor by the uterine sound. In case of failure by all the preferred methods, delivery may always be secured by puncturing the membranes. In doing this the liquor amnii may be saved in part by making the opening into the membrane four or five inches above the os. A male catheter is used, with a truncated extremity, through which a silver wire may be protruded to make the puncture.—*Memphis Med. Rec.*

